# Tweeting Supertyphoon Haiyan: Evolving Functions of Twitter during and after a Disaster Event

**DOI:** 10.1371/journal.pone.0150190

**Published:** 2016-03-28

**Authors:** Clarissa C. David, Jonathan Corpus Ong, Erika Fille T. Legara

**Affiliations:** 1 Graduate Studies, College of Mass Communication, University of the Philippines, Quezon City, Philippines; 2 Department of Media and Communication, University of Leicester, Leicester, United Kingdom; 3 Complex Systems Group, Computing Science Department, Institute of High Performance Computing, A*STAR, Singapore, Singapore; Université Toulouse 1 Capitole, FRANCE

## Abstract

When disaster events capture global attention users of Twitter form transient interest communities that disseminate information and other messages online. This paper examines content related to Typhoon Haiyan (locally known as Yolanda) as it hit the Philippines and triggered international humanitarian response and media attention. It reveals how Twitter conversations about disasters evolve over time, showing an issue attention cycle on a social media platform. The paper examines different functions of Twitter and the information hubs that drive and sustain conversation about the event. Content analysis shows that the majority of tweets contain information about the typhoon or its damage, and disaster relief activities. There are differences in types of content between the most retweeted messages and posts that are original tweets. Original tweets are more likely to come from ordinary users, who are more likely to tweet emotions, messages of support, and political content compared with official sources and key information hubs that include news organizations, aid organization, and celebrities. Original tweets reveal use of the site beyond information to relief coordination and response.

## Introduction

The social network microblogging site Twitter has played an increasingly important role in communication during disasters, both on the side of the public and of institutions involved in disaster relief and response. In parallel, the literature on functions of Twitter during disasters is growing, with different disciplines examining unique aspects of its use in hazardous events. Research shows that it is most heavily used as a platform to disseminate information quickly through a population, to a lesser extent as a source of information from people who are witnesses to an event, and as a way for the public to contribute to efforts in generating awareness through retweeting of relevant information [1–5]. As our knowledge continues to develop about how Twitter is used during emergencies, and how its purposes can be expanded, users continue to innovate on its functions before, during, and after a disaster.

This paper contributes to current knowledge by showing how the content of Twitter during and after a global humanitarian event evolves over time, from a channel of information dissemination about a storm and its impacts, to a venue for mobilizing relief and response on a global scale, to a place to where emotions are shared. Content analyzed Twitter messages related to Haiyan, over the course of 20 days, suggest that the issue attention cycle [6] on Twitter for this global humanitarian crisis began to wean after 9 or 10 days. Unlike much of the existing work in this area we analyzed retweeted messages and non-retweeted messages separately, allowing for a differentiated view of the site’s functions for those who compose original posts (most likely ordinary people) and those that are widely shared across the network, which often are from official sources and key information hubs. This way results are not heavily skewed toward the types of messages that are most often shared, and the voices of ordinary Twitter users who are not often retweeted are not drowned out in the analysis. Results indicate that there are meaningful differences in the content and sources of tweets that are the most-retweeted, and the ones that emanate from the broader audience. While there has been interest in the use of Twitter during crises [7, 8], this paper contributes to this growing body of work by focusing on how attention cycles operate in social media platforms and identifies the voices and messages that drive attention and conversation.

Twitter data were collected the day before supertyphoon Haiyan (locally known as Yolanda) and for 18 days afterwards. Haiyan was a trending topic on Twitter for over two weeks, with activity coming from many countries. This was the strongest typhoon to ever make landfall in recorded history [9, 10], severely damaging areas of different countries in Southeast Asia. It directly hit the middle of the Philippine archipelago, which is comprised of a group of islands, decimating the city of Tacloban in Leyte province and flattening many other municipalities along its path. While both local and international relief efforts were quickly mobilized from outside the province, it took several days for help to reach the affected areas. For over a week little was known about the extent of the damage, the situation on the ground, where survivors were, and how many were killed (government figures report over 6,000 people died in Tacloban, but thousands more remain missing one year after Haiyan hit). Delayed access to the site also perhaps lengthened the attention cycle on social media and traditional media, as information about damage in Tacloban was slow to reach the press because of difficulties in reaching affected areas. The international media were on site as soon as transportation systems allowed, broadcasting to the world images of devastation, stories of loss, and as days passed with no help arriving, criticisms against the national government’s efforts rose.

### Twitter’s functions during disaster events

While Twitter was not originally conceived for use in emergency response situations and disaster events, the public and institutions are increasingly turning to it for gathering and disseminating information [11]. The growing empirical work on Twitter’s functions in relation to disasters shows that it is a valuable channel of information for both official institutional sources such as government agencies and news outfits, and witnesses on the ground close to the event who are able to post updates as texts and photos. Its use has been studied in relation to the 2011 Tohoku earthquake [12] the Red River Valley flood threat [4], the Pakistan floods [1], the Waldo Canyon wildfires [13] the Australian floods [14] and several others. Twitter contains features that are particularly useful for disaster reporting and monitoring: real-time posting, short-burst message style (140 characters), default public settings when posting, multimedia capacities, hyperlinking, presentation of posts in reverse chronological order, and easy retweeting or forwarding of messages to followers and the public Twitter space.

Informal networks on social media are used for different purposes, including information transmission, emotional expressions, activity reporting, media content sharing, and collective sensemaking [11, 15, 16]. During emergencies Twitter functions as a backchannel source of information [17], a means of rapid information dissemination [8], a venue for information sourcing to enhance situational awareness [18], and as a channel for sharing opinions and experiences [19, 20]. There may be other functions as yet understudied, following Palen et al’s [4] observation that Twitter and other social media serve various functions depending on the type of user and type of event.

A number of studies have closely examined various aspects of Twitter’s use as an information source and channel for dissemination during disasters. In addition to being a place to find and share information, people also use it to share personal experiences, crack jokes, express concern, and ask questions [19]. Information sharing is done often through retweeting of links, with a larger portion of links coming from news websites rather than official government sources [21, 22, 23]. For many studies of Twitter messages during disasters, the main finding is that over half are information-related and contain links to websites (e.g. Refs. [24] and [19]). The platform functions largely as a broadcasting medium during emergency events and other non-routine situations [24].

In response to its institutional use as a broadcasting medium, people or “audiences” use the site to actively seek out information—a more common behavior than starting an online conversation or other similar forms of interactive strategies [25]. Publics are drawn to the site by active information seeking because in crisis situations, affected populations seek out explanations to reduce uncertainty. Information “hubs” [26, 27] tend to be official sources with announcements and situation updates, dominating the most retweeted messages and reaching the widest audiences. A content analysis of Twitter messages posted by international organizations involved in the response after Haiti’s earthquake revealed evolving use of the medium over time [28] from that of information dissemination, to expressions of support, to fundraising. The earthquake was a trending topic on Twitter for several weeks, throughout breaking news, fundraising, organizing of events, and updates on response on the ground [29].

Engaging through Twitter as a response to disasters and crisis situations is motivated by a felt “need to contribute, and by doing so, (being) better able to cope with the enormity of the situation” [17] (p.5). A study of people who tweeted about Haiti found that their reasons included connecting with others for a common cause, promoting relief efforts, and personalizing their own activities in response to the disaster [30]. From an organization’s point of view, nonprofits and media institutions craft messages on Twitter to motivate citizen response [31]. This in turn generates greater interest in taking action like donating money or contributing to relief efforts.

In light of these previous findings, we coded for many different types of content using the Twitter posts during and after Haiyan. These include information about the typhoon and its damage, posts about disaster relief as reported from the ground, reports of different disaster relief efforts from those who are initiating them, posts of emotional response (e.g. “afraid of Haiyan”), supportive messages (e.g. “sending prayers for Tacloban”), and posts about politics. This will allow for a detailed view of the make-up of Twitter posts overall throughout the issue cycle of Haiyan, and changes across the days in terms of content.

### Global interest in disasters

The impact of Haiyan was hardest on a handful of islands in the Philippines, but the interest in the typhoon, the damage it created, and the recovery of hardest hit towns like Tacloban, was international in scale. A day after the Philippine President Aquino declared a “State of Calamity” and formally accepted international assistance on 11 November 2013, the United Nations declared a Level 3 humanitarian response and triggered relief operations from international governments and NGOs [32]. Countries like Japan, the US, Australia, and the UK pledged billions of dollars in aid funds and sent valuable disaster experts and military personnel in disaster response. Humanitarian agencies descended on the small city with emergency supplies, bringing their own transportation, communication, and fuel. Closely following the responders were international media such as CNN, NBC and other television networks in the US, the BBC of the UK, the wires, and many others from different countries. Sentiments on Twitter came from around the globe, with messages conveying support, emotion, and sharing of efforts to contribute to the relief operations. This was an event that held the attention of many people in many countries, similarly with other global humanitarian crises like the Haiti earthquake, the Japan tsunami, and the Pakistan floods.

Global interest in local crises may be more common in the age of online social networks. Following the predictions of Castell [26], information flows in the digital world now reflect structures of networks organized around communication hubs and nodes. Communication hubs function as main exchangers; coordinating elements in the network while nodes become the bases for locally initiated activities (p. 443). Content can be generated by any person or organization, and shared to or by any entity on the network. This communication structure, enabled by Twitter, allows local citizens to broadcast worldwide and allows the world to respond. During Haiyan, information flows with such patterns was evident for many days. Photos of hard-hit areas were being tweeted by relief workers on the ground, and then shared across the world. The global public posted messages of support and in some cases, used the platform to coordinate response.

The 2010 Haiti earthquake, in many ways, brought into global focus the capacities of Twitter for disaster outreach and fundraising [31]. Scholars have pointed out how humanitarian agencies recognized the significance of Twitter for aid mobilization in the context of Haiti [33, 34]. One organization successfully designed a communication protocol to have mobile SMS systems and Twitter messages work together to make donations to Haiti relief efforts convenient. The NGO ARC, used a 5-digit number to text if a person wants to donate, and then used Twitter to publicize the mechanism for donating in a fixed $10 amount [35]. In eight days $305 Million was raised [28, 36]. As humanitarian agencies continue to develop innovative social media approaches to fundraising and response, they will become a greater presence in Twitter traffic during and after disasters.

Murthy and Longwell’s [1] examination of the uses of Twitter during flooding in Pakistan suggests that there is a global/local divide in perceived legitimacy of information. While most links shared by Twitter users emanated from traditional media with social media trailing far behind, social media sources were more often linked to, among those tweeting from Pakistan. Moreover, messages from Pakistani sources were conferred more authority status during the floods, a manner of communication from sites of disasters that were previously unavailable in events of global interest. Social networking sites are now members of the larger media ecosystem that operates during disasters of global implications; they have become an important part of the communication system used to inform organizations and citizens [37].

In a communication environment where online social networks, no matter how transient, provide an “always on” firehose of news and information from many places to many places, certain types of events like disasters will find its way more easily to global consciousness. When information sources are as diverse as they are on Twitter, the source becomes an important heuristic in sorting out good information from bad. Official information sources like governments and news organizations would be the entities with the widest reach and following [22, 38] as their status confers credibility onto information tweeted. Language matters as well, primarily as an indicator of where the source is located and who their main audience is. Local-language tweets (local to the country of the disaster event) may have different content from English-language tweets intended for a global audience. Local users may produce and seek out particular content from their media, especially for those in the disaster zone. In contrast, English-language tweets may be written with a global audience in mind and express solidarity through more general language.

The evolution of content, dominant sources, and volume of Twitter traffic post-disaster is reflective of an issue cycle for similar events on the social network site. Communities that form are informal and loosely knit together, those who are part of the Haiyan community are held together by a series of hashtag terms. Most of these connections disappear eventually several days following an event. It is posited that this issue cycle, the one that will be found for Haiyan, would be similar with what one would find for any large-impact disaster event that finds a global audience.

### Gaps in the literature and research questions

The growing literature on Twitter use during disasters paint a consistent picture of its value and function as an expeditious way of disseminating information. Information often emanate from official sources, mostly news organizations. Many of these studies however, do not examine changes of tweeted content over time, although there are those that offer observations of overall declines in information sharing as days pass. Since retweets comprise a large portion of overall Twitter traffic during disasters, this means that keeping the retweets and original posts together will obscure non-informational types of content that regular citizens may be posting. The analysis in this paper separates these two categories of messages and compares patterns between them.

We expect that by coding for other message content that are not purely information, namely posts about: disaster relief, emotion, support, personal experience, and political commentary, a more detailed understanding of the various functions of Twitter during such events can be discussed. To illuminate whether and how content evolves over time and the potential alternate forms of content that are posted, we ask:

RQ1: How do volume and content of Twitter messages about a disaster evolve over time?

RQ2: Are there differences in message content between the most widely shared messages and original posts on Twitter?

Following research on the news issue attention cycle (e.g. Refs. [6] and [39]), a longitudinal analysis of the rates of increase and decrease of various types of messages about Haiyan on Twitter should provide valuable information on parallel issue attention cycles in relation to global disasters. Coding for other types of content like emotions and supportive messages may reveal differing patterns from informational posts, when examined through the days post-disaster.

To provide a more nuanced understanding of the network’s behavior regarding reception and sharing of certain types of messages, features of the most retweeted messages were analyzed for content and source to determine characteristics of the most retweeted messages.

RQ3: Which types of content are most shared? What types of messages and sources act as the most effective communication hubs on Twitter during disasters?

## Method

Tweets were collected using a Twitter API at the “Spritzer” level between November 7 and 27, 2013. This means that 1% of all tweets posted are made available by Twitter to the API for collection. The tweets were filtered to include messages that contain the following keywords: BagyoPH, RescuePH, FloodPH, TracingPH, ReliefPH, TyphoonHaiyan, HaiyanPH, HaiyanPH, Haiyan Typhoon, Typhoon Haiyan, Haiyan, TyphoonPH, Mindanao, Visayas, Samar, Leyte. These include both the international and local name of the typhoon, names of places in the Philippines that were hit, common hashtag words for the typhoon, and official hashtags used by the Philippine government over Twitter. In addition to the message tweeted, the data included user location for a subsample, Twitter handle, time zone of the user, hashtags, time the tweet was posted, whether the tweet is a reply to a message, and whether a tweet is a retweet. All Twitter data collected for this study are publicly available and can be collected by anyone in the same manner as it was collected here. The data collection was conducted in compliance with the terms and conditions stipulated by the site. The fields included Twitter usernames alone which are publicly viewable, and dates of the post, data collected did not include any identifying information or geolocation tags. There are no data protection committees in the Philippines that may be consulted, but data collected were not shared with anybody outside of the authors and research assistants named in this paper.

During large natural disaster events (e.g. Queensland floods, Haiti earthquake) much of the circulating information are retweets from media and other official sources. These predominate the universe of tweets and are often the focus of many published studies since the contents of retweeted messages are considered the most influential. This research focuses as well on the content of unique messages being tweeted, to provide greater visibility in the data toward individuals that post thoughts and information, the “public” as it were. Thus, the dataset were first filtered of retweets, this was done in an automated manner that removes all messages beginning with “RT.” The most retweeted messages were analyzed separately. After the automated filtering, which excluded a large proportion of the total Twitter data (70%), two undergraduate students manually scanned the dataset to delete duplicates that did not have RT tags, messages that were not in English or Filipino, and messages that were not related to Haiyan. These deletions comprised 35% of the dataset without RTs. The process yielded a dataset that reflects original comments by different sources and represents 19.5% of the original set of tweets from the Twitter feed API collection (n = 536,363). From the cleaned dataset, a random sample of 1,000 tweets was drawn per day, resulting in a final sample size of 20,000 (S1 File for full data availability statement).

Two graduate students coded the sample for a number of variables that indicate the content of tweets. Krippendorff’s kappa was calculated for each variable to test for reliability, with each round of reliability testing done with a sample of 500 tweets from the full dataset minus retweets. Acceptable reliability levels according to De Swert [40] are .80 and above, with a minimum of .67 for exceptional circumstances. The majority of coded variables reached the .80 acceptable threshold (S1 Table) with a few exceptions. Variables with reliability coefficients between .67 and .79 had low incidence rates and were therefore very sensitive to each disagreement. The table reports percent agreement and incidence for these variables.

The coding scheme was designed so that all content variables could overlap; no restrictions were placed for mutual exclusivity. That is, a single tweet could contain typhoon information, emotional expressions, and a comment about climate change and would then be coded for each of the three. In making decisions, they were instructed to read the body of the text as well as all the words with hashtags (#). Some tweets would have content-relevant words in hashtags, for instance a tweet with #heartbroken or #sad would be coded under negative emotion.

The most retweeted messages were coded in an identical manner. For each day of data collection, data were ranked according to the number of times a message was retweeted. The top 100 most retweeted messages for each day were compiled and coded using the same coding scheme and by the same coders. Sources of the most retweeted messages were also coded into categories that included news media, Philippine government sources, international aid organizations, and media celebrities.

### Measures

Appendix A contains all content variables coded, their definitions, subcategories, examples, and reliability coefficients. Two background variables were coded, language and source. Since the research team has the capability to comprehend the top 2 languages used in the dataset during Haiyan, these were tagged for purposes of comparative analysis of content by language. These languages were Filipino or Tagalog and Cebuano (and closely related dialects). Source was coded in a centralized manner, a list of all unique Twitter handles was generated and a graduate student did research-based determinations of whether the handles belonged to private individuals, entertainment media celebrities, heads of institutions (e.g. Pontifex as head of Catholic Church), politicians, aid organizations, and local or international media.

The remaining content variables fall under the following general categories: information, expressions of support, emotion, disaster relief and aid, and political expressions. Each category has a number of subcategory variables defined and described below:

#### Information

These tweets contain information about the typhoon, its impact, and where to help with monetary donations. Although solicitation of information does not happen often, these were coded as well. There are a number of sub-categories of information coded, to allow for disaggregation in the analyses when there is an interest in specific types of information. *Typhoon information* is tweets containing information about the weather phenomenon of Haiyan itself, including trajectory, strength, time of landfall or place of likely impact. *Class suspension* was coded separately; these are simple announcements about suspension of school and work as a result of the typhoon and its damage. *Damage information* are messages that describe the extent and type of damage, number of deaths, places hit, or scale of the impact. *Fundraising information* are shared details about drives to raise money to send to victims of the storm. *Requests for information* include queries about current situation or specifics like whether an area was hit, whether a street is flooded, or whether electricity has been restored.

#### Disaster relief

In the aftermath of the typhoon institutions and individuals undertook massive relief and recovery efforts. Tweets that were about disaster relief were coded in several subcategories. *Personal relief* are activities of any relief effort a tweeter has provided to the affected populations. These include monetary and nonmonetary aid. *Relief of others* is those that report efforts of other people and institutions. *Relief coordination* is messages that contain actionable information pertaining to the provision of aid. For example, tweets that say what a community needs, or where one can go to volunteer, or messages containing logistical information reporting on the status of aid distribution. *Specific calls for help* contain specific information like place where help is needed and what kind of help is needed (e.g. please send water to Guian, people are in dire need of help).

#### Foreign response

Many tweets report on the pledges of support from countries and international aid agencies, even individuals in places outside the Philippines. These were coded into a separate category and include news about monetary or nonmonetary assistance provided by any entity that is not based in the Philippines. Foreign response may overlap with disaster relief, and in those cases the tweets would be coded positively for both. The distinction would reveal when the event reached and sustained international attention.

#### Personal information

Messages that contain personal reports and questions were tagged. These include messages that ask for information to *help finding people* (e.g. looking for any news about my sister, was Alangalan Leyte badly hit?), specific *local information about impact* or status of the storm (e.g. please send help to Bantigue Leyte, there are people there dying of hunger), reports of direct *personal loss* of people or material possessions (e.g. our house in Tacloban is completely gone). Personal information is separated out as its own category to try to distinguish tweets from those who were directly affected or witnesses, from those that are general information from institutions such as government and media.

#### Emotions

The literature on use of Twitter suggests one of its main functions is for users to tell others how they feel. Emotions were coded into three broad categories: *negative*, positive, and *expressions of gratitude (thanks*). Messages of thanks were pervasive over some days, and thus, were coded separately from other forms of positive emotions, however in the end positive emotions were not reliably coded. Since there are only few instances of positive emotion tweets, the reliability tests were extremely sensitive to even the slightest disagreement. However, since we were coding for negative emotions it would be inappropriate to not code for positive ones. Results for positive emotions should be interpreted with caution. Negative emotional statements included expressions of shock, sadness, grief, and anger among others. Expressions of positive emotions included having hope, feeling inspired.

#### Expressions of support

Haiyan was a global phenomenon both as a weather disturbance and after it hit Tacloban, as a humanitarian crisis. We coded for messages that expressed *calls for safety* (e.g. stay safe everyone in the Philippines!), and *prayers* (e.g. praying for everybody in the path of the supertyphoon). A separate category on other *supportive messages* was coded but did not achieve reliability (.57) and are thus, excluded in the analyses.

#### Politics

Discussions and news about politics and government were common in the days after the storm hit, related to relief and recovery operations. These were primarily criticisms related to the slowness of response, to statements made by public officials, and news and commentary about fights between political factions (local and national government). Positive and negative messages were coded in separate categories to capture valence.

The data were examined longitudinally to reflect changes in functions over time. Simple descriptive summative statistics are used to provide an overall picture of the types of messages that are posted related to Haiyan. By-day distributions of variables are presented to examine longitudinal patterns. By-group and by-day differences presented are statistically significant, a result that is more readily attained since the sample size is large.

## Results

**Fig 1:**
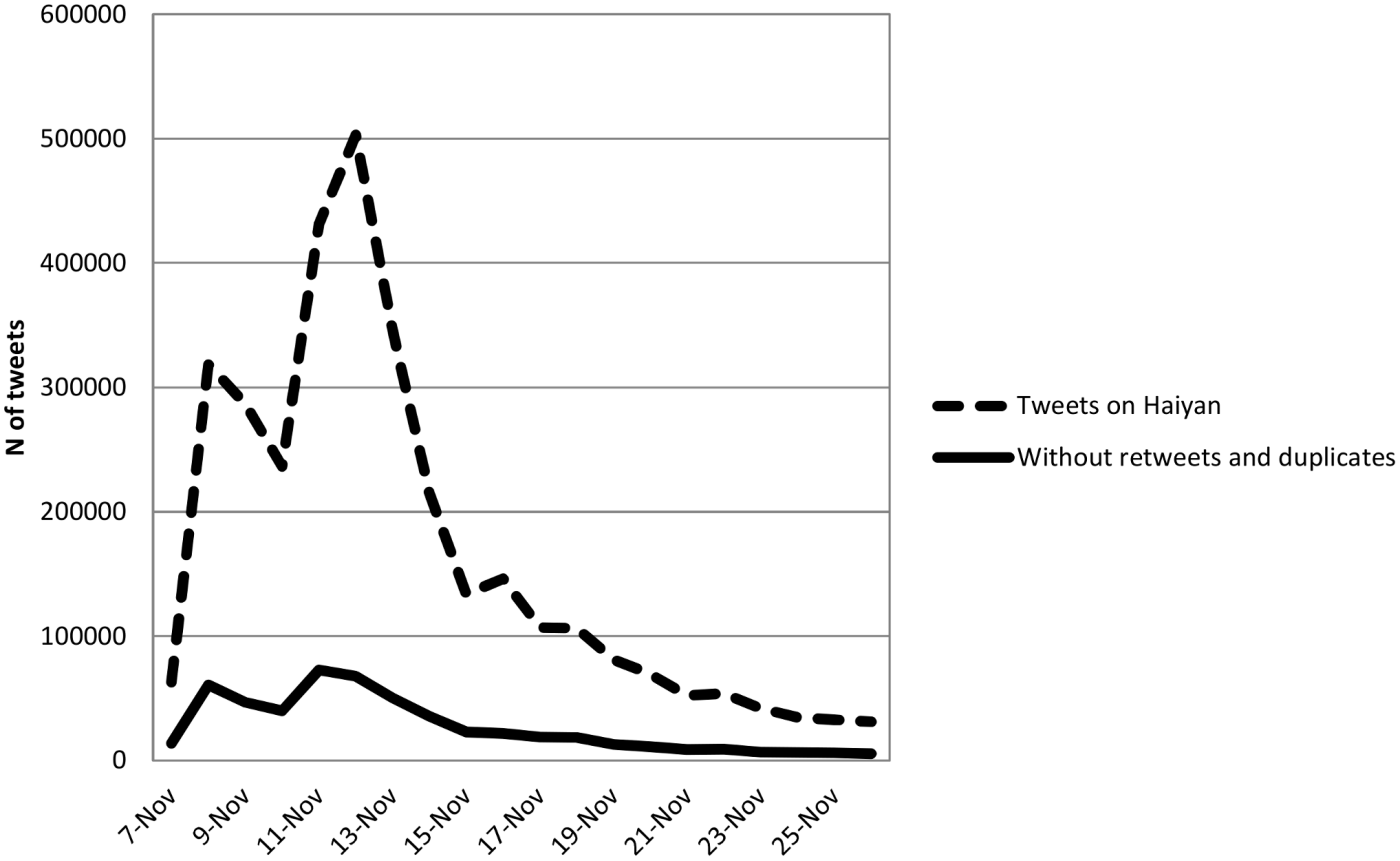
Number of tweets by day.

The first research question is about how volume and content of Twitter messages about a disaster evolve over time. Fig 1 shows the number of tweets collected by day through the API, since these are a fixed percentage, randomly selected sets of tweets from the collected data, it is assumed that the volume changes per day in the sample reflect volume changes per day among all collected tweets. Tweets about Haiyan started off November 7 with low frequency, shooting up to 5 times its volume the on November 8; the day the storm hit Tacloban at 5 in the morning. While the storm was happening and the day it made landfall, there was little information coming out of the hardest hit areas. Towns were isolated from any communication and transportation for close to 48 hours, reflected in a slight dip in tweet volume following November 8. Tweets increased again starting November 11, peaking on 12th. Tweets during these days were about the scale of the damage, the press had arrived on site and descriptions, photos, and videos were being circulated to the public. The rate of decline in tweet volume was equally steep in the following two days. Thus, from this example the issue attention cycle peters off after about 17 days.

The vast majority of tweets were retweets, a manner for individual citizens to share information they see on the platform. The solid line in Fig 1 illustrates volume after all retweets are excluded, showing a much flatter line, although an increase in original content posting is also evident following the same dates as the line including retweets. Differences in patterns of overall Twitter traffic and posts that exclude retweets reflect the platform’s use as an effective dissemination channel. Through communication hubs and social networks, information can be retweeted any number of times.

**Fig 2:**
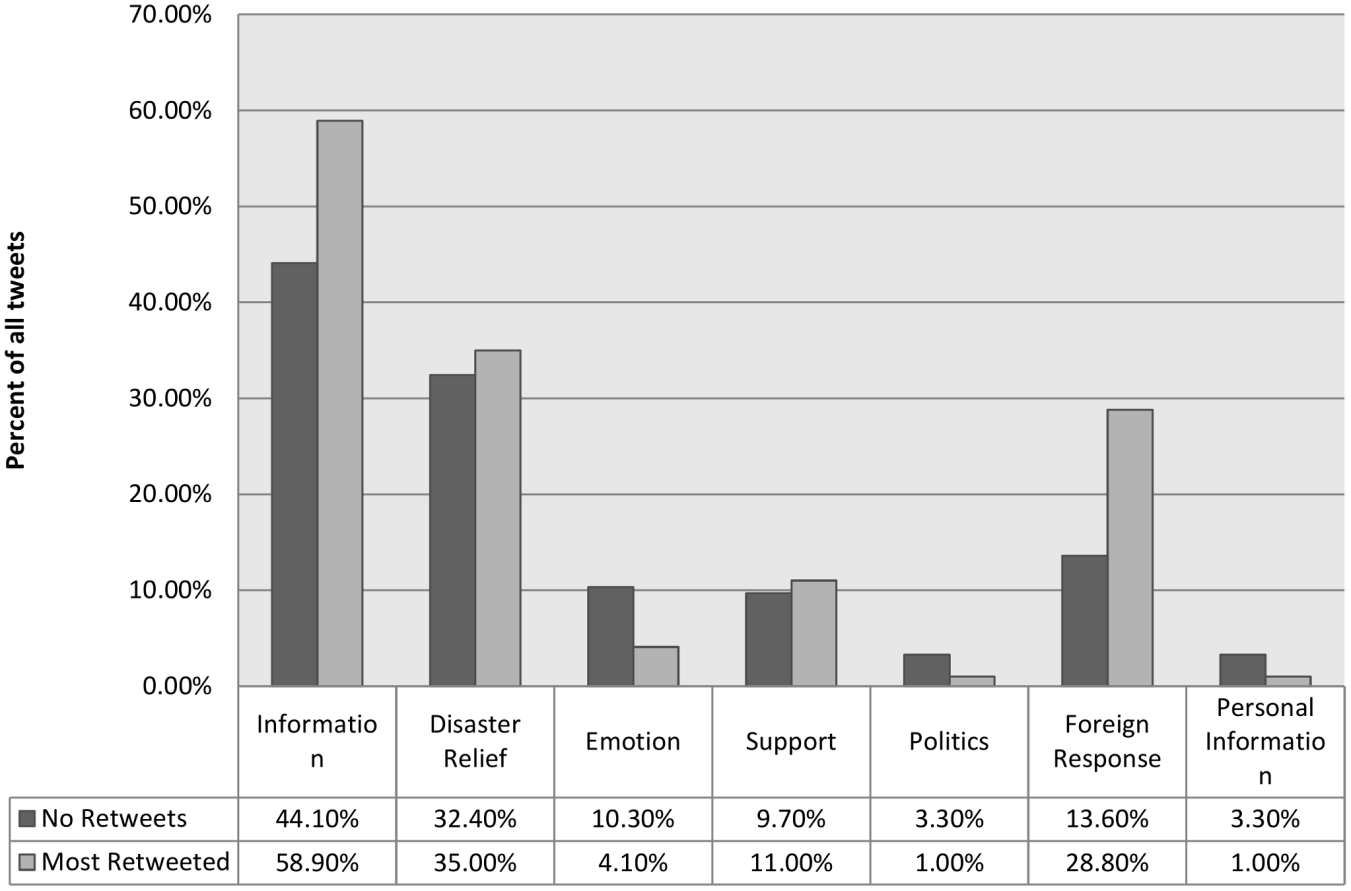
Content of tweets.

Much of the available literature on Twitter posts during disasters find that the majority of content being posted is information, by a large margin, with other forms of posts lagging far behind in frequency. The second research question asks whether there are differences in message content between the most widely-shared messages and those that are original posts. Fig 2 shows general content category distributions for both the most retweeted messages and messages without retweets. While 59% of the most retweeted messages contain information about the typhoon, a smaller portion (43%) of the general Twitter messages contains information. Still, the overall pattern is that information about the typhoon and about disaster relief dominates Twitter. We argue that the vast majority of original posts are from ordinary citizens or the general public. Results show that these tend to contain more messages with emotions, personal information, and commentary about politics, although these do not occur frequently. Among the original tweets, 10% of messages have content about emotions, compared to a smaller 4% among the most retweeted messages. Personal information in general does not get tweeted often, however given the scale of Twitter [41], even small proportions could be consequential. There are more instances of personal information tweets from original posts compared to the retweeted messages.

Of note is a dearth of messages related to politics. According to an international comparative study by Pew Research Center [42], while social media enjoys relatively high use rates in the Philippines, its use for posting content related to politics is low compared to other developing countries. This is reflected in the content coding done here, where political posts comprised very little of the total Twitter traffic on Haiyan. This could be specific to the country however, and it would be interesting to do further cross-country comparisons of Twitter use during global humanitarian crises to determine if the lack of political discourse post-disaster varies by location.

**Fig 3:**
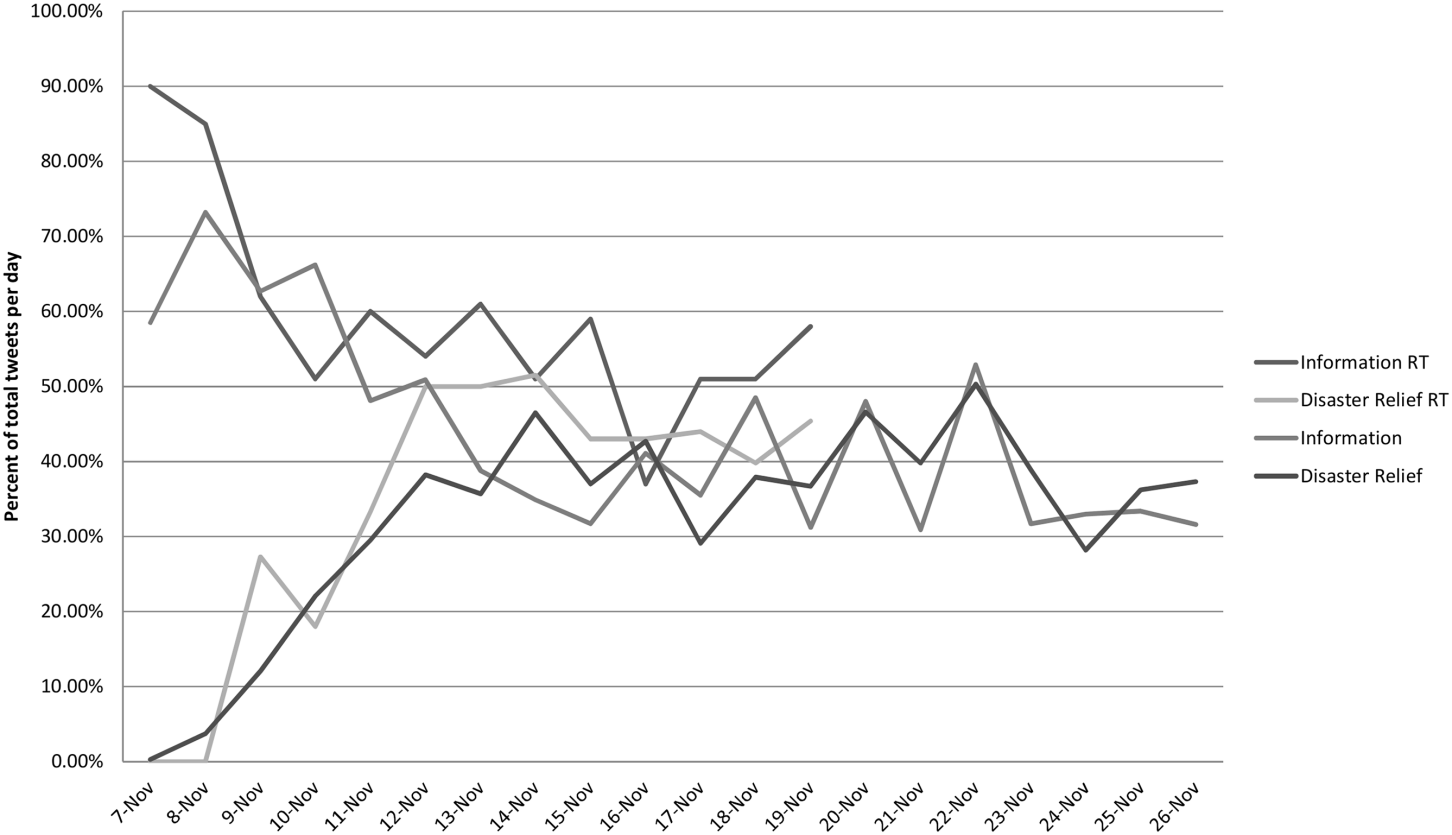
Tweets of information and disaster relief.

Over the course of several days, tweets with informational content about the typhoon and its damage declines sharply for both retweets and non-retweeted messages (Fig 3). These are replaced by messages with information about disaster relief and foreign response (which often also contain information about relief; 34% of disaster relief messages are about foreign response). As a proportion of total tweets these two categories comprise the vast majority of posts. Emotions are shared across all the days for tweets of the general public, peaking at 16.8% of tweets the day the storm hit, with the lowest values at 5% at 20 days post-typhoon. For retweets, emotions do not comprise a substantive proportion of messages, hovering around 3% for the days around the typhoon, and then peaking 8 days after at 10%.

Informational tweets among the general public are comprised of 18% information about the typhoon, 8% about fund drives, 4% questions asking for information, and the vast majority, 75% information about the damage and impact of the typhoon (categories are not mutually exclusive). The volume of informational tweets decreases over time but always stays above other forms of content in terms of proportion of total tweets.

Of interest are tweets about disaster relief, as these have not been examined as a separate category in existing research on the functions of Twitter during disasters. Of the total sample of tweets from the general public that were classified under disaster relief, 12% reported on personal acts of disaster relief, 67% reported on the relief activities of others, 8% were solicitations of specific kinds of help (e,g. water needed in Guian, Samar. It’s been 3 days), and 54% are coordinative in nature (e.g. @abscbnnews: find your family in #Haiyanph-hit areas using the #kapamilyafinder: http://t.co/9oxy6trvkt (click "find"; @reliefphcom please add abs-cbn legaspi to the list of drop-off points in albay. #reliefph #rescueph; pia-ndrrmc: doe has 147 generator sets waiting to be transported to leyte, eastern samar, roxas capiz). Twitter, therefore, not only acts as a channel to disseminate information about the typhoon and the disaster relief activities that go on, it also is used as a platform to coordinate and mobilize relief efforts, to share personal stories helping, and to share information about help being extended by others.

**Fig 4:**
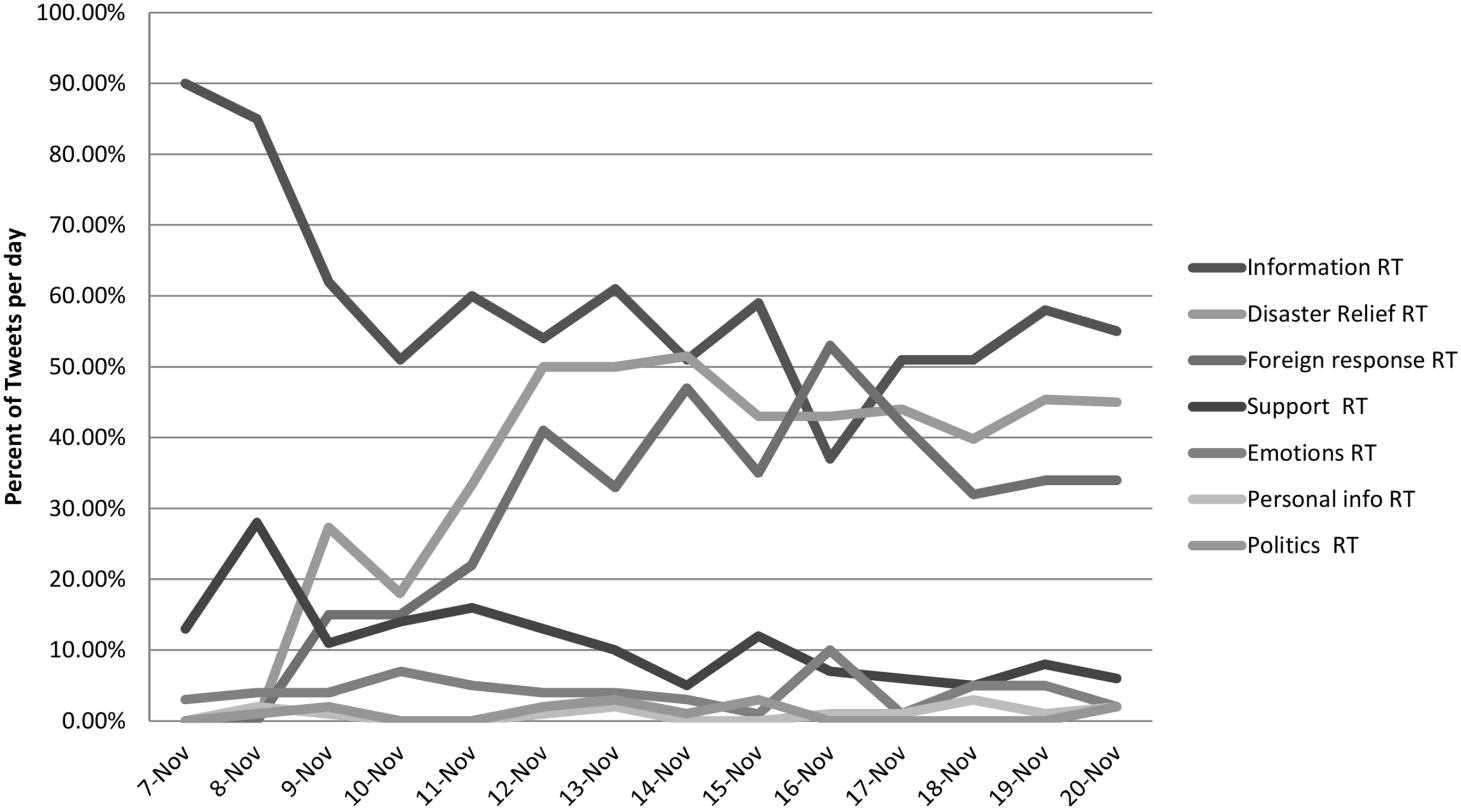
Content categories by day, retweets.

Retweets are mostly information messages for more than a week after the typhoon, comprising 85% and 90% the day of the typhoon and the day before it, respectively (Fig 4). These are messages with news about the intensity and size of the storm, its trajectory, and where it will make landfall. There are large increases in news about the foreign humanitarian response to those affected, starting at 15% only 2 days after, and rising to 53% of the most retweeted messages one week after. Disaster relief messages that circulate among the most retweeted messages are usually about large fundraising campaigns, these began in earnest the day immediately after the storm, the day that information about the size of its damage started appearing on the news. By November 12, 50% of the most retweeted messages were about disaster relief, staying at around that level until the end of data gathering.

There are slight by-language differences in the overall distribution of content types tweeted by the general public. Filipino language tweets, which comprises 11% of the sample (English 87%), have higher rates of messages with emotions, support, politics, and information (Table 1). English language tweets have higher rates of messages about disaster relief and foreign response. Local-language tweets represent sentiments of people who are closer to the site of the disaster and therefore are either directly or indirectly affected, as opposed to the rest of the world on Twitter who are spectators to the event as it unfolds. The international community responded through disaster relief, and this is evident in the higher proportion of disaster relief messages in English.

**Table 1:** Content of non-retweeted messages by language (percent).

	Filipino	English
Information	53	42.5
Disaster Relief	22.9	31.9
Foreign Response	5.9	13.2
Personal Information	8.3	2.8
Emotions	18.5	9.3
Support	13.8	10.2
Politics	5.8	2.7

**Fig 5:**
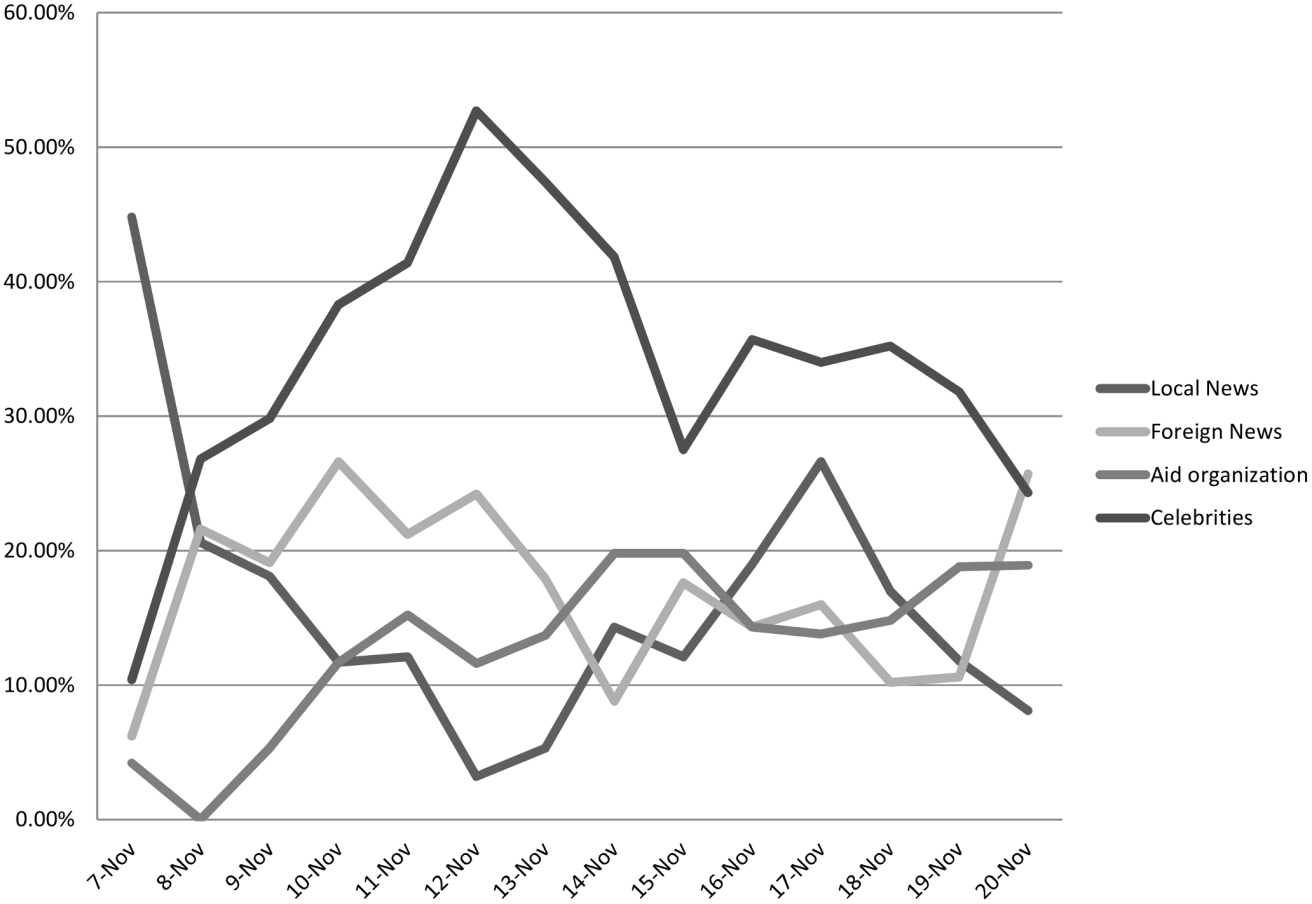
Sources of most retweeted messages, by day.

The most retweeted messages are often from news sources, media celebrities, and aid organizations involved in disaster relief. Fig 5 shows the progression of how different sources of tweets dominate in different stages of the issue cycle. Interestingly, while we coded for Philippine government agencies and key officials, these were not substantive sources of popular information on Twitter. In the initial days when circulating messages are comprised mostly of information about the storm and the extent of damage, local news sources played an important part in circulating critical safety messages. Their influence over the network began to decline as soon as news about the scale of the damage became public, international news organizations started covering it as a global event. Foreign news sources had more retweeted messages than local news sources two days after the storm and continued for a week. Local news became more prominent again beyond November 16, when local news outfits carried a story about the Empire State Building having been lit up as a tribute to those killed by Haiyan. The most influential communication hubs are clearly celebrities, most of whom are non-Filipino celebrities. Table 2 lists the top most retweeted messages each day, and for most days the top are from celebrities Harry Styles, Liam Payne, and Alicia Keyes. The current Pope Francis, head of a state and a religious institution, we argue, has celebrity status given his high visibility in international media. Celebrities clearly have an inordinate amount of influence over circulating information on the network, particularly during disasters when they are active in publicizing fundraising efforts. Aid organization are also a prominent source of the most retweeted messages, these are mostly fundraising drives and are aided in turn by the activities of interested celebrities.

**Table 2:** Most retweeted messages, top for a sample of days.

Day	Tweet	N of RT
7	@makatiinfo: dear students: instead of praying for class suspension, pray for the safety of those who are in the direct path of	976
9	@pontifex: i ask all of you to join me in prayer for the victims of typhoon haiyan / Haiyan especially those in the beloved is	13768
11	@aliciakeys: to the people of the philippines my heart is with you #praye	3413
13	@harry_styles: millions in the philippines need clean water & shelter now. @oxfamgb is there: donate to their #haiyan appeal! h	27187
14	@harry_styles: millions in the philippines need clean water & shelter now. @oxfamgb is there: donate to their #haiyan appeal! h	5580
16	@empirestatebldg: 2nite & tmrw night, our lights shine in the color of the #philippines flag 2 raise awareness of the #haiyan t	6410
17	@harry_styles: millions in the philippines need clean water & shelter now. @oxfamgb is there: donate to their #haiyan appeal! h	1639
19	@real_liam_payne: contribute to #haiyan relief efforts. donate to the @redcross via itunes at http://t.co/	8063
20	@real_liam_payne: contribute to #haiyan relief efforts. donate to the @redcross via itunes at http://t.co/	5578

**Fig 6:**
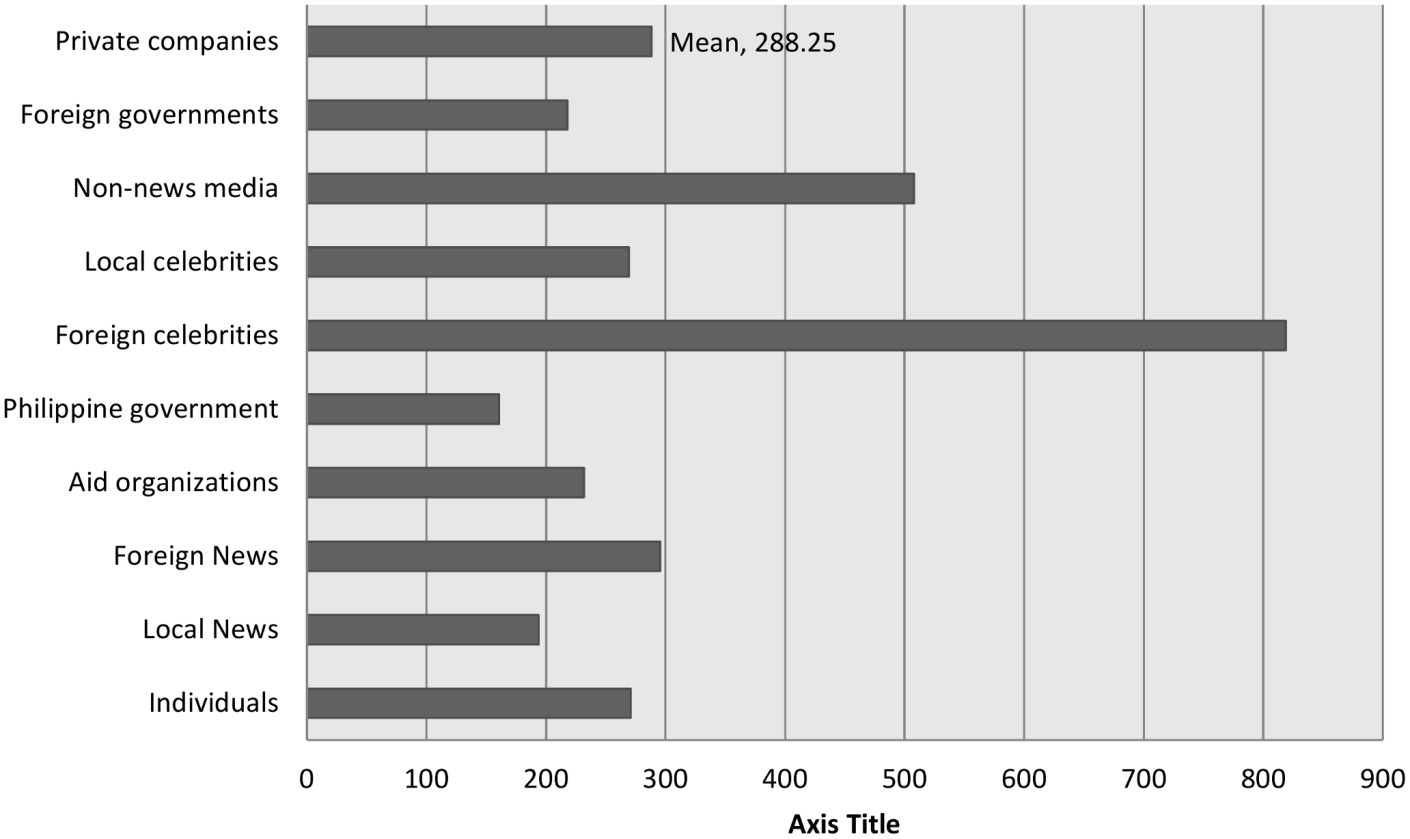
Average number of retweets by source.

To answer research question 3, even more so than news organizations, media celebrities act as the most effective communication hubs for disseminating across the network, if we define effectiveness as being able to enable the widest dissemination. Fig 6 shows the average number of retweets by source among the most retweeted messages, clearly indicating a large advantage of foreign (non-Filipino) celebrities over all other sources. Interestingly, non-news media sources are the second most-retweeted sources, these are often social media-centric Twitter accounts like Instagram or television brands like National Geographic and the Ellen DeGeneres Show.

## Discussion and Conclusions

This research sought to provide an over-time examination of the various functions of Twitter, reflected by the types of messages that are posted, how these message types gain dominance and then decline in frequency, the main communication or information hubs in the network and how their posts differ from those of the general Twitter public. By examining these throughout a 20-day period between the day before Haiyan, a predicted weather event, to the days when rescue and relief were in full force, we hope to show a more nuanced picture of the issue attention cycle on the Twitter network. This is shown in the context of a global weather event that turned into a humanitarian crisis, since it hit a developing nation. As the biggest storm in recorded history hit the Philippine island of Leyte, killing more than 6,000 people with a 20-foot storm surge, Twitter users posted information, retweeted, and engaged actively in the relief effort.

Consistent with findings of studies that examine social media use during disasters, posts related to Haiyan were comprised mostly of retweeted informational content [8, 43]. About 80% of the total traffic in the 20-day span were retweets of messages from media sources, celebrities, and Philippine government agencies. As a proportion of tweets, retweets comprised the largest portion of messages on the day the storm hit, and 5 days afterward. When news stories become available, users take it upon themselves to retweet throughout their network. The steep curve of information dissemination on Twitter suggests that users are not just relying on the site for news but also have a sense of engagement, enough to retweet stories that are of interest. Changes in volume of retweets indicate the public and news’ engagement with the issue, which declined sharply on the 5^th^ day and then slows even more over the next few days. This, we propose, is a typical news issue cycle on Twitter, and with similar studies of other global disaster events may be reflected in other instances. An event’s presence on the site may not be as extended for disasters that do not have international attention, as much of this attention was sustained by disaster relief efforts by organizations that are not in the Philippines.

Without the retweets in the dataset, the content analysis reveals that original tweets coming from ordinary people are of only a slightly different character in terms of content type distribution. Ordinary people are more likely to tweet emotional expressions, reports about disaster relief efforts, and personal information. While the differences do not appear large compared to the retweets, and the proportions are at or below 10%, the massive scale of Twitter’s traffic [41] means those even low proportions could translate to tens of thousands of messages. These types of messages have not been examined thoroughly in the literature, possibly because retweeted messages usually are included in sample codings, drowning out alternative types of messages being posted by the regular users. The exception is Chew and Eysenbach [19], but their study is in the context of a global pandemic and its interest is more in Twitter’s use as an alternative source of statistical information for a disease’s spread. In the case of global disaster events such as Haiyan, which evolves into a humanitarian crisis, the types of posts from ordinary people and their changes over time indicate Twitter’s functionality as a space to express solidarity and work through emotions. Significantly, it is also a space where people share information and activities about disaster relief done by those outside of the Philippines. Such information sharing and locally organized fundraising and volunteering carry great potential for government and humanitarian agencies to widen their pool of potential volunteers and donors.

The role that Twitter plays as a functional communication tool for disaster preparedness, emergency response, crisis communication, and relief provision continues to grow as institutions and individuals innovate on the various ways it can be used to deploy and gather information. Disaster relief messages comprised a substantial portion of both the retweets and general public messages. The retweeted messages are mostly announcements and retweets of celebrities that publicize fundraising drives of aid organizations. However other tweets that are not among the most retweeted contain actionable coordinative information. These include things like calls for volunteers to a specific location to help with packing goods for delivery to affected sites, calls for volunteers with cars to drive relocated individuals to places where they can stay, and lists of supplies needed in specific locations. These are important logistical coordinative functions that may spur greater involvement among those who are active on the Twitter platform. Beyond the functionality for publicity during fundraising activities, Twitter may also effectively improve upon disaster response and relief delivery from volunteers and aid groups insofar as it brings together into conversation engaged and interested actors. Twitter amplifies voice, creating a potentially wider audience, greater mobilization efforts, and greater attention from the aid community at large as disaster events capture global attention more easily now than before. Nevertheless, we recognize that it is important to also assess the impact of Twitter in relation to how affected people themselves accessed and used Twitter and benefited from any campaigns organized through the platform. Research on affected communities’ own uses of social media suggest a disconnect between global initiatives (which we identify here as having many potential benefits for fundraising and aid mobilization) with experiences on the ground [44].

The evolution of posted content moves from a predominance of information about the typhoon to disaster relief messages and reactions and stories about foreign response. The news cycle, media sources writing stories about the storm and its aftermath, sustains attention at a high level for several days. This signals the relevance of official information sources in providing the original content that generates interest in the network, as it is retweeted at scale, attention spreads throughout different countries bringing the event to international importance. This high level of attention, indicated by the volume of retweets in the early days, creates a community of followers of hashtags about Haiyan which in turn serves as the main audience for all other non-informational posts that follow in the days of response and relief. As members of the Twitter public contribute to relief efforts in various ways (e.g. donating, joining events, attending concerts, volunteering services, being directly involved in aid), they post about their own and others’ efforts, extending issue interest in the network and potentially creating a sense among followers of the hashtags that a large public is directly involved, which may in turn create a perceived social normative pressure to contribute [45].

Celebrities had a central role in acting as information hubs and key entities in the network that would increase attention and then sustain it through time. Specifically, Harry Styles and Liam Payne, members of a musical group called One Direction with a large international fan base, showed up in the results as being central information hubs for disaster relief drives. There are many other celebrities that appear among the top sources of retweeted messages, highlighting the importance of information hubs in creating wide networks of followers for issues. Even the Twitter fan pages of celebrities often appear as top sources of retweeted content on certain days. The scale of this effect is large, and a systematic empirical network analysis of the size and speed of information diffusion from celebrities is in order to clarify exactly the depth and breadth of this influence.

This research is limited by some key constraints. First, the spritzer level access to Twitter data and the opacity of the selection of tweets the platform supplies limits the ability of many studies like this to show the real scale of attention and content diversity. However, this does not threaten the validity of the distributions and relationships examined here. Second, the content analysis can only be done for English language and Filipino language tweets, excluding many messages that were posted in other foreign languages that are a substantive portion of the international attention. Third, the sheer volume of even sampled content made coding of sources difficult to complete, as information is not available for every Twitter account that was sampled. This limited our ability to draw inferences about the relationship between content types and sources for the non-retweeted messages.

Use of Twitter during and after disaster events spans a variety of functions including information dissemination, disaster relief and response coordination, fundraising, and emotional expression. The distribution of content in the network evolves in the days following a disaster event, particularly one that becomes a global humanitarian issue as what happened in Haiyan. Various sources play important roles as information hubs that either generate original content, or in the case of celebrities, amplify the reach of messages to new publics that would otherwise be less engaged and aware. The issue attention cycle on Twitter, taking into account key sources and hubs, and the evolution of content, provides important information for strategic communication. Crisis communicators from government can apply what is learned here to widen the audience base for messages. Aid organizations doing fundraising drives should connect with effective amplifiers of messages such as entertainment celebrities to improve collections. Smaller microvolunteer networks in local areas can use it to broadcast needs and coordinate effective response.

News outlets can use Twitter accounts to sustain attention on the issue by continued postings of new information constantly, even if the information base changes from details about the event to details about the response. It would be of empirical and theoretical interest for future research to examine whether the issue attention cycle on Twitter is different from that of other media outlets, or even for that matter, from Facebook.

Unfortunately, global weather events that lead to humanitarian crises are a constant threat and will continue to happen periodically. Efficient and targeted use of available communication channels such as social media networks can make a real difference in preparing communities for disasters, and transforming response and relief into larger and more effective efforts.

## Supporting Information

S1 File Full data availability statement. (DOCX)

S1 Table Coding categories, definitions, examples, and reliability coefficients. (DOCX)
