# Supplementary material for: Tweeting Supertyphoon Haiyan: Evolving Functions of Twitter during and after a Disaster Event
Source: PLoS One. 2016 Mar 28;11(3):e0150190. doi: 10.1371/journal.pone.0150190 (PMC4809515; doi:10.1371/journal.pone.0150190)
Supplement: S1 File — (DOCX) [file pone.0150190.s001.docx]

Full Data Availability Statement

Partial data will be made available for download, excluded fields are Twitter names and content of posts. Full data are available upon request. Raw data include Twitter handles (account names) and posts, sometimes posts contain full names of private individuals. The data captured by the research team were collected at the point in time of posting, if users deleted their posts with full names of individuals, these cannot be verified or deleted from the research data files.

The Twitter dataset was obtained using one of Twitter's Public Streams ([http://dev.twitter.com/streaming/public](http://dev.twitter.com/streaming/public)) that supply researchers with samples of the public data flowing through Twitter via its Streaming API ([http://dev.twitter.com/streaming/overview](http://dev.twitter.com/streaming/overview)), which, in turn, allows access to a global stream of tweets. We set up a listener (a machine that collects streaming tweets via public stream--at the "spritzer access" level) that was coded in Python, allowing us to establish a connection to the Twitter's Public API. The tweets were collected using the request parameter "track" where we listed a set of keywords related to the topic at hand. The collected tweets were automatically stored in a database via an SQL database engine.

For the exact dataset in this research, interested researchers may need to use Twitter's Search API ([http://dev.twitter.com/rest/public/search](http://dev.twitter.com/rest/public/search)) instead of its Streaming API since we are now dealing with historical tweets. However, for the Search API, Twitter imposes rate limits ([http://dev.twitter.com/rest/public/rate-limiting](http://dev.twitter.com/rest/public/rate-limiting)); more importantly, "the Search API is not complete index of all Tweets, but instead an index of recent Tweets," which makes it impractical to collect the same dataset (more than 2 years old) via Twitter's Public API. One option is to subscribe to one of Twitter's special permission/access (e.g. via Gnip - [http://gnip.com/sources/twitter/)](http://gnip.com/sources/twitter/))."
