# Supplementary material for: Tweeting Supertyphoon Haiyan: Evolving Functions of Twitter during and after a Disaster Event
Source: PLoS One. 2016 Mar 28;11(3):e0150190. doi: 10.1371/journal.pone.0150190 (PMC4809515; doi:10.1371/journal.pone.0150190)
Supplement: S1 Table — (DOCX) [file pone.0150190.s002.docx]

Coding categories, definitions, examples, and reliability coefficients

| Category | Subcategory (K alpha) | Definition | Example |
| --- | --- | --- | --- |
| Language | Language (.85) |  |  |
| Source |  | Category of tweet sender | Government, media, celebrity etc |
| Information | Typhoon information (.89) | Tweets containing information about the typhoon or weather phenomenon. These include information such as trajectory, time of landfall, windspeed. | @cnnbrk: haiyan may be strongest cyclone to hit land ever recorded: sustained winds of 195 mph, gusts to 235 mph |
|  | Class suspension (.8) | Suspension of classes AND work, including questions |  |
|  | Damage/Impact information (.85) | Descriptive messages about extent and type of damage, including casualty counts (deaths injuries). | death toll rises in philippines, 153,495 hectares (ha) of rice paddy, maize and other high value crops such as coconut, banana and vegetables have been hit by haiyan |
|  | Fundraising information (.83) | Information about fundraising drives | last day for rhian ramos' #fansignforacause. have a fansign from rhian and donate at the same time #Haiyanph |
|  | Requests for information (.81) | questions about the current situation or about specifics like whether an area is damaged, whether a street is flooded, whether electricity has been restored | @gmanewsonline may mga relatives po ako sa jaro, leyte and still i'm worrying about their status now |
| Emotions | Negative emotions (.8) | General expressions of sadness, grief, shock and other similar negative emotions as a result of the typhoon, its impact, the relief effort and others. | i was watching the tv and i cant help but cry.it really breaks my heart :( #prayforphilippines #rescueph #bangonpinoy |
|  | Expressions of sympathy (.71) | Messages that show support or sympathy towards victims of the typhoon | our hearts go out to the victims and families of typhoon haiyan |
|  | Thanks (.78) | Any message that expresses thanks, gratitude, appreciation directed toward aid providers, government, general others | cant thank every1 enaf that helped the victims of typhoon haiyan! thank you all so much! u are all wonderful!god bless u |
| Disaster Relief | Personal relief activity (.74) | Personal reports of help the tweeter has provided to others, actions done to respond and provide relief/help/aid | i will donate my savings for almost 4months for the victims of Haiyan |
|  | Relief activity of others (.69) | Posts that report/describe relief activities that are being conducted not by the person posting, including those done by groups, other individuals, aid agencies, government etc. | fatburger family collecting donation and relief goods for the victims of super typhoon haiyan, unhcr is sending aid packs and tents for #typhoon #haiyan victims in #philippines |
|  | Relief coordination (.85) | Disseminating information about Needs for relief goods, transportation etc. Includes calls for donations, questions asking how they can help | :@dswdserves opens new repacking hubs in metro manila.volunteers also needed in other regions, ateneo dswd repacking station needs 80k bags by sat. other dswd stations are lasalle, are there relief operations in Ateneo right now? |
|  | Specific calls for help (.75) | particular information about the kind of help that is needed or where | <name> - tanauan, leyte - - who can help <name>, from saudi arabia |
| Foreign response | (.67; 15/500 cases in reliability coding) | Messages that focus on foreign response or aid. These refer to BOTH countries AND AID agencies (e.g. oxfam, world bank). | China pledges 100 thousand dollars. US sending marines. |
| Personal information | Help finding people (.9) | Messages calling for help finding specific people missing or out of contact | @gmanewsonline please po kung pwede po puntahan ninyo ang pinamopoan,capoocan leyte nag aalala na po kami.tabi lang po kasi sila ng dagat |
|  | Direct personal loss (.73) | Information on surviving relatives, finding relatives and friends, all must be personal. This includes messages about friends of friends, friends of family, family of friends, and others that are not "generalized" | i cant contact my families at leyte today. their mobile phones are all unattended. #prayer |
| Support | Calls for safety (.82) | Messages calling for people to stay safe, specific or general | Stay safe everyone |
|  | Prayers (.84) | Messages callng for people to pray, including any messages that refer to God and use the term "bless" and other references to religion | Praying for the safety of those in the path of Haiyan |
| Politics | Criticism of Philippine government (.69) | Negative assessments and expression of negative emotions about the Philippine government, officials, agencies and politicians | these filipino politicians are just a bunch of leeching, soul-less fucktards. i wish you experienced the wrath of #Haiyanph too |
|  | Positive assessments of government (.67:2/500 cases in reliability coding) | Positive assessments and expression of positive emotions about the government, officials, agencies and politicians |  |
|  | Media performance (.67; 3/500 cases in reliability coding) | Tweets that comment on the manner in which media is covering the typhoon and its aftermath | i am really proud of @atomaraullo he helped the victms of typhoon Haiyan, he don't mind being soaked in flood water.this man is a true hero |
